# Minimizing dose variation from the interplay effect in stereotactic radiation therapy using volumetric modulated arc therapy for lung cancer

**DOI:** 10.1002/acm2.12264

**Published:** 2018-01-25

**Authors:** Kazuki Kubo, Hajime Monzen, Mikoto Tamura, Makoto Hirata, Kentaro Ishii, Wataru Okada, Ryuta Nakahara, Shun Kishimoto, Ryu Kawamorita, Yasumasa Nishimura

**Affiliations:** ^1^ Graduate School of Medical Sciences Department of Medical Physics Kindai University Osaka Japan; ^2^ Department of Radiation Oncology Tane General Hospital Osaka Japan; ^3^ Faculty of Medicine Department of Radiation Oncology Kindai University Osaka Japan

**Keywords:** interplay, number of breath, SBRT, VMAT

## Abstract

It is important to improve the magnitude of dose variation that is caused by the interplay effect. The aim of this study was to investigate the impact of the number of breaths (NBs) to the dose variation for VMAT‐SBRT to lung cancer. Data on respiratory motion and multileaf collimator (MLC) sequence were collected from the cases of 30 patients who underwent radiotherapy with VMAT‐SBRT for lung cancer. The NBs in the total irradiation time with VMAT and the maximum craniocaudal amplitude of the target were calculated. The MLC sequence complexity was evaluated using the modulation complexity score for VMAT (MCSv). Static and dynamic measurements were performed using a cylindrical respiratory motion phantom and a micro ionization chamber. The 1 standard deviation which were obtained from 10 dynamic measurements for each patient were defined as dose variation caused by the interplay effect. The dose distributions were also verified with radiochromic film to detect undesired hot and cold dose spot. Dose measurements were also performed with different NBs in the same plan for 16 patients in 30 patients. The correlations between dose variations and parameters assessed for each treatment plan including NBs, MCSv, the MCSv/amplitude quotient (TMMCSv), and the MCSv/amplitude quotient × NBs product (IVS) were evaluated. Dose variation was decreased with increasing NBs, and NBs of >40 times maintained the dose variation within 3% in 15 cases. The correlation between dose variation and IVS which were considered NBs was shown stronger (*R* ^*2*^ = 0.43, *P *<* *0.05) than TMMCSv (*R* ^*2*^ = 0.32, *P *<* *0.05). The NBs is an important factor to reduce the dose variation. The patient who breathes >40 times during irradiation of two partial arcs VMAT (i.e., NBs = 16 breaths per minute) may be suitable for VMAT‐SBRT for lung cancer.

## 1 INTRODUCTION

Stereotactic body radiation therapy (SBRT) using volumetric modulated arc therapy (VMAT) has been widely investigated in recent years.^1^, ^2^ Verbakel et al.^1^ showed that SBRT using VMAT allows delivery of hypofractionated doses over much less time than conventional SBRT using 10 static noncoplanar fields, with the additional advantage of the plans being more conformal compared with those in conventional SBRT in peripheral stage I lung cancer.

VMAT‐SBRT is, however, susceptible to dose variation from the interplay effect between the multileaf collimator (MLC) sequence and tumor motion.^3^ The dose variation may not be negligible in VMAT‐SBRT, which is generally completed with a few fractions. Jiang et al.^4^ showed that the maximum dose variation due to the interplay effect can be up to 30% for one intensity modulated radiation therapy (IMRT) field over one fraction, and 18% for all five IMRT fields over one fraction. Court et al.^5^ showed that dose error could be >5% for the area of 40% in the target when target motion was 2 cm for VMAT plans (2 Gy/1 fraction). Tyler et al.^6^ found that VMAT‐SBRT deliveries showed an increased interplay effect with maximum deviation of ±4.8% in dose received at least 1% (D1%) of the gross tumor volume (GTV). It is important to improve the magnitude of dose variation that is caused by the interplay effect. The number of breaths (NBs) during irradiation was focused on an improving factor for the dose variation in this study. The aim of this study was to investigate the impact of NBs to the dose variation for VMAT‐SBRT to lung cancer.

## 2 METHODS AND MATERIALS

### 2.A Patient selection

The data of 30 consecutive patients who underwent treatment with VMAT‐SBRT for lung cancer between July 2011 and July 2015 at our institution were selected. Of these 30 patients, 17 patients were primary lung cancer and 13 patients were metastatic lung cancer. The tumor location, amplitude of respiratory motion in the craniocaudal direction, and respiratory cycle are summarized in Table 1.

**Table 1:** Descriptive statistics of patient characteristics and plan parameters

Case	Tumor location	Amplitude (cm)	Respiratory cycle (sec)	Total MU	Irradiation time (sec)	NBs^a^ (times)	MCSv^b^	TMMCSv^c^	IVS^d^
1	Left upper	0.8	2.9	1643	164.0	56.7	0.409	0.511	14.50
2	Left upper	0.8	3.3	1723	172.0	52.2	0.428	0.535	13.97
3	Left lower	1.6	5.0	1446	145.0	28.9	0.492	0.308	4.45
4	Right upper	0.3	4.5	1878	188.0	41.7	0.363	0.725	17.63
5	Right upper	0.3	4.5	2128	213.0	47.3	0.326	1.087	25.68
6	Left upper	0.4	3.7	1816	182.0	49.1	0.381	0.953	23.43
7	Left upper	0.3	2.2	1369	137.0	62.2	0.467	1.556	52.68
8	Left upper	0.3	2.2	1579	158.0	71.8	0.421	1.403	50.37
9	Left upper	0.8	2.5	1489	149.0	59.5	0.462	0.578	17.17
10	Left upper	0.8	2.5	2517	252.0	100.7	0.284	0.355	17.89
11	Left upper	0.2	3.0	1145	115.0	38.2	0.538	2.691	51.35
12	Right middle	0.4	3.0	1435	144.0	47.8	0.490	1.225	29.33
13	Right middle	0.4	3.0	2226	223.0	74.2	0.294	0.734	27.27
14	Left lower	0.3	4.1	1232	123.0	30.0	0.586	1.953	29.34
15	Left upper	0.3	2.7	1926	193.0	71.3	0.355	1.184	42.23
16	Left lower	0.3	2.3	1559	156.0	57.7	0.418	1.394	40.25
17	Left lower	0.3	2.3	1770	177.0	65.6	0.366	1.219	39.95
18	Left lower	0.5	3.8	1973	197.0	51.9	0.369	0.739	19.19
19	Left upper	0.5	3.8	1953	195.0	51.4	0.345	0.691	17.82
20	Left upper	0.2	5.2	1524	152.0	29.3	0.505	2.523	36.94
21	Left lower	0.2	5.2	1933	193.0	37.2	0.376	1.878	34.91
22	Left lower	0.7	2.7	1949	195.0	72.2	0.376	0.537	19.39
23	Left upper	0.7	2.7	1851	185.0	68.5	0.396	0.566	19.39
24	Left upper	0.6	2.5	2134	213.0	85.4	0.309	0.515	21.97
25	Left upper	0.6	2.5	2206	221.0	88.2	0.297	0.495	21.84
26	Left upper	0.3	3.4	2034	203.0	59.8	0.325	1.084	32.47
27	Left upper	0.3	3.4	1976	198.0	58.1	0.351	1.171	33.99
28	Left upper	0.2	3.4	1934	193.0	56.9	0.339	1.696	48.23
29	Left lower	1.1	3.7	1223	122.0	33.1	0.521	0.473	7.82
30	Left lower	1.1	3.7	1703	170.0	46.0	0.365	0.332	7.65
Mean		0.5	3.4	1776	178.0	56.4	0.398	1.037	27.3
Maximum		1.6	5.2	2517	252.0	100.7	0.586	2.691	52.7
Minimum		0.2	2.2	1145	115.0	28.9	0.284	0.308	4.45
Standard deviation (1 SD)		0.3	0.3	329	33.0	17.8	0.08	0.631	13.45

a Number of breaths during irradiation.

b Modulation complexity score applied to VMAT.

c the MCSv/amplitude quotient.

d the MCSv/amplitude quotient × NB product.

### 2.B Treatment planning

Four‐dimensional computed tomography (4DCT; GE Medical Systems, Waukesha WI, USA) was employed with breathing phases identified by an infrared marker and camera system (Real‐time Position Management System (RPM); Varian Medical Systems, Palo Alto CA, USA). For each patient, 10 three‐dimensional computed tomography (3DCT) images, corresponding to equally spaced phases of a respiratory cycle, were reconstructed from 4DCT images and imported into a treatment planning system (TPS; Eclipse ver. 10, Varian Medical Systems, Palo Alto CA, USA). The amplitude of each tumor trajectory was assessed by measuring the peak‐to‐peak tumor position from the phases of the breathing cycle with the TPS.

The GTV was contoured on lung window level CT images over all breathing phases by an oncologist. The GTVs were then merged to generate the internal target volume (ITV) on the average CT image which was reconstructed from the 4DCT. A planning target volume (PTV) was created by adding an isotropic margin of 5 mm around the ITV. In all plans, the prescription dose was 70 Gy delivered in 10 fractions,^7^ with at least 95% of the PTV being covered by the prescribed dose. The dose was calculated based on the average CT image using the anisotropic analytical algorithm with inhomogeneity correction. A dose calculation grid size of 2.5 mm was used.

Plans were derived using a NovalisTx linear accelerator (Varian Medical Systems, Palo Alto CA, USA) equipped with a high‐definition (HD120) MLC (2.5 mm leaf width in the central region). All VMAT plans used 6 MV and were delivered in two partial arcs (0°–180°, clockwise and counterclockwise) to avoid the contralateral lung. Collimator angles of 30° and 330° were used for each arc to reduce the cumulative effects of interleaf transmission and the tongue‐and‐groove effect. The created treatment plans were actually used to treat the patients.

### 2.C Patient specific quality assurance

#### 2.C.1 Dose measurement

Dose measurements were performed with a micro ionization chamber (PinPoint ionization chamber 0.015 cm^3^; PTW, Freiburg Germany) in a Quasar phantom (Modus Medical Devices, London ON, Canada). The ionization chamber was placed in the center of a cylindrical cedar insert (to represent lung density), which can undergo cyclic movement in the craniocaudal direction with user‐specified amplitude and movement pattern. For static measurements, the ionization chamber was aligned at the isocenter. The measured doses then were compared with recalculated TPS data on a static CT image of the phantom. Dynamic measurements were performed using an amplitude and respiratory waveform of the patients’ 4DCT scan data, repeated 10 times for each plan. The 10 measurements were performed with random starting phases of the breathing cycle. The measured doses were compared with the mean dose recalculated with TPS on each phased CT image of the phantom. These phased CT images were reconstructed from 4DCT image which was acquired with the phantom moved by the amplitude and respiratory waveform. Dose calculation was performed with inhomogeneity correction and the grid size was 2.5 mm. The standard deviation calculated from the results of 10 dynamic measurements was defined as dose variation due to interplay effect.

#### 2.C.2 Dose distribution

The dose distributions were also verified with radiochromic film (Gafchromic EBT3 ISP, Wayne, NJ, USA) to detect undesired hot and cold dose spot. The film was placed in the coronal plane through the isocenter of the cylindrical insert. While the cylindrical insert moved according to the respiratory waveform and amplitude of each patient, measurements were performed twice with different starting points of the respiratory cycle. EBT3 films were scanned at least 24 hr after irradiation with an Epson ES‐10000G flatbed scanner (Seiko Epson Corp., Nagano Japan). Images were acquired in transmission mode and landscape orientation. RBG images were collected at a depth of 16 bits per color channel with a spatial resolution of 300 dpi and were saved in .tiff format.^8^ The two dose distributions were aligned by localizing the films using installed room laser before the irradiation. And then, they were compared with dose profile and gamma passing rate using RIT 113 version 5.4 (Radiological Imaging Technology, Colorado Springs CO, USA) as first measured dose distribution was a reference. A dose difference of 2% and a distance to agreement of 2 mm were selected for the gamma analysis to evaluate regions receiving at least 30% of the maximum dose.^9^

### 2.D Plan parameters

To assess the relationship between dose variation and factors related to the interplay effect, quantitative analysis of parameters related to respiratory movement of the tumor and the complexity of the MLC sequence was performed. The complexity of the MLC sequence was evaluated using the modulation complexity score for VMAT (MCSv) introduced by Masi et al.^10^ The MCSv has values in the range of 0–1. The value of MCSv decreases with increasing MLC sequence modulation. MCSv was calculated from the Digital Information and Communication in Medicine Radiation Therapy (DICOM‐RT) files using in‐house software. As mentioned above, the amplitude for each tumor was derived from the phases of the respiratory cycle with the TPS. In addition, the NBs during irradiation of two partial arcs with VMAT were assessed for each patient. The NBs during irradiation can reflect both the irradiation time and respiratory cycle. The NBs during irradiation were calculated from the following equation:

(1) $$NBs=\sum_{i=1}^{I}\left(\frac{irradiation\;\;time_{i}}{T}\right)$$

where *I* is the number of arcs in the plan, and *T* is the mean time of a single respiratory cycle. The mean time of a single respiratory cycle in each patient were obtained from RPM system. *Irradiation time* was calculated from the dose rates and the monitor units (MU) at each control point of the plan. Therefore, irradiation time was assumed that machine is operating at the optimal dose rate for the entirety of the treatment.

The examinations were assessed to verify dose variation affected by NBs as follows: (a) While changing NBs (the mean respiratory periods were changed by multiplying by factors of 0.5, 1.3, and 2.0), the doses were measured with the micro chamber for the same plan. Consecutive 16 patients were used in 30 patients. Dose variations calculated from measurement with three different NBs were compared with that in the original NBs. (b) Two indices combining the MCSv, amplitude, and NBs were introduced. The magnitude of interplay effect cannot be evaluated from only one factor, because the interplay effect is caused by combination of various factors such as amplitude, breathing cycle, dose rates, and MLC sequence.^4^, ^5^, ^6^ Therefore, the combination of tumor motion and MCSv (TMMCSv) is calculated as the quotient of the MCSv and amplitude. A smaller MCSv implies a more modulated/complex plan, and it was assumed that more modulated plans can produce more interplay. A smaller amplitude may cause a smaller interplay, therefore the value of the MCSv divided by the amplitude was smaller for a more dose variation.

(2) $$TMMCSv=\frac{MCSv}{A}$$

where *A* is amplitude. TMMCSv is assumed that the value is closer to 0 according to the smaller dose variation. The other index (IVS: interplay effect variable score) is calculated as the product of TMMCSv and NBs. It was assumed that undesired hot and cold dose spot can be improved by a larger NBs.

(3) IVS=TMMCSv×NBs

Likewise, IVS is assumed that the value is the closer to 0 according to the smaller dose variation.

The parameters obtained from all plans were analyzed with descriptive statistics. The correlations between the dose variation and the described NBs, MCSv, TMMCSv, and IVS were evaluated using the coefficient of determination (*R* ^2^). Statistical analyses were performed using IBM SPSS Statistics software, version 22 (IBM Corp., Armonk NY, USA).

## 3 RESULTS

### 3.A Case‐specific QA

**Figure 1:**
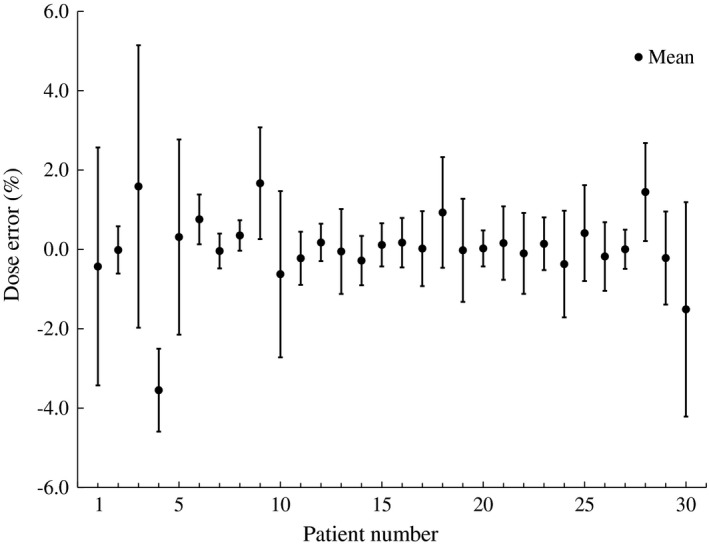
The differences between calculation dose and measurement dose, with dose variation calculated from the dynamic measurements for each patient. The error bars show one standard deviation for each patient.

The absolute doses measured by static measurement were maintained within 3% of recalculated TPS data on a static CT image of the phantom in all plans, with an average of −1.2 ± 0.63%. Figure 1 shows the dose errors for each patient using the ionization chamber. The dose variations were from ±0.4% (patient number 8) to ±3.6% (patient number 3).

**Figure 2:**
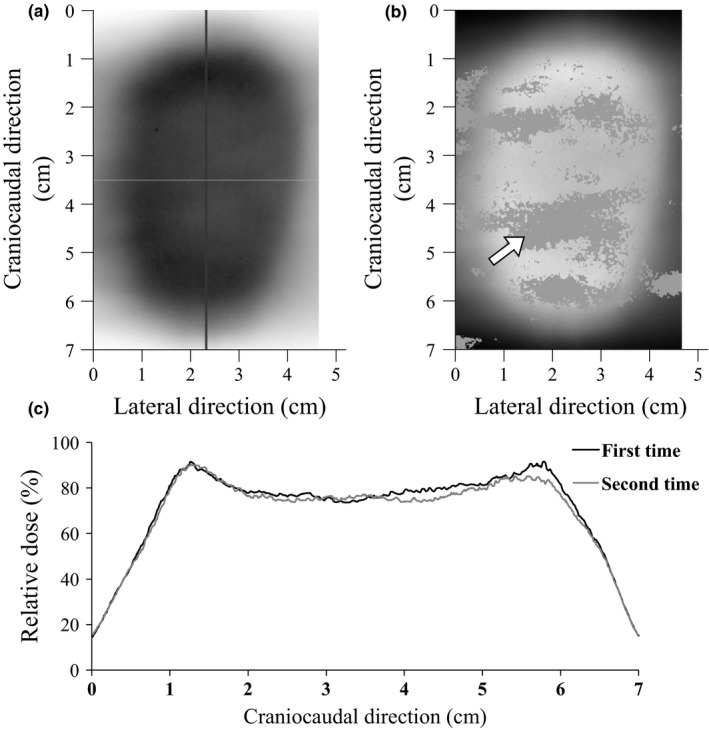
Comparison between measured dose distributions in patient number 3. (a) film image, (b) result of γ evaluation method (tolerance values; 2%/2 mm, threshold 30%), (c) dose profiles in craniocaudal direction through the isocenter. Film measurements were performed twice for each patient. The dose distributions were obtained during irradiation while moving the phantom with the patient's respiration waveform and amplitude along craniocaudal direction. Measurements were repeated two times with random starting phases of the breathing cycle. In the γ evaluation method, the area with a γ value >1 appears in gray. The white arrow shows the point of the maximum dose difference (10.4%). In the dose profile, the black solid line indicates the first measurement, and the gray solid line indicates the second measurement.

An example of comparison between dose distributions that were obtained in patient number 3 while operating the phantom with patient's respiratory waveform and amplitude during irradiation is shown in Fig. 2. Figure 2(b) shows the result of the γ evaluation method (tolerance values; 2%/2 mm, threshold 30%), and Fig. 2(c) shows the dose profiles of both dose distributions in craniocaudal direction. The dose variation was extensive, and the maximum dose difference was 10.4% [white arrow in Fig. 2(b)].

### 3.B Plan parameters

The mean MCSv was 0.4 ± 0.1 (range, 0.28–0.59). The NBs during irradiation was an average of 56.4 ± 17.8 times (range, 28.9–100.7 times). Patient number 3 had 28.9 NBs during irradiation (total irradiation time 144.6 s), with about 12 breaths per minute, which was the lowest recorded among the 30 patients. Mean values of TMMCSv and IVS were 1.05 ± 0.63 (0.31–2.69) and 27.7 ± 13.3 (4.4–52.7), respectively.

**Figure 3:**
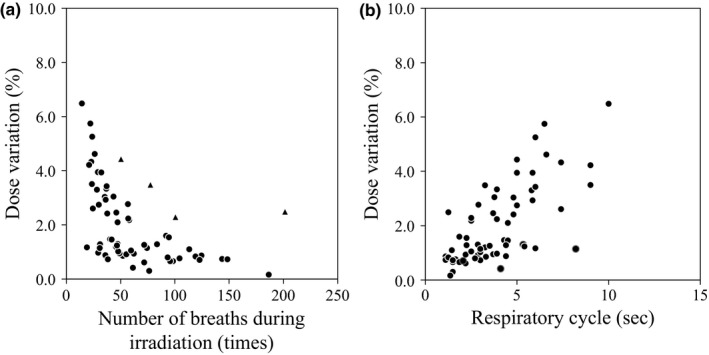
Dose variation measured with different number of breaths in the same plan. (a) Dose variation vs. number of breaths during irradiation. Closed circles show results of patient number 1–9 and 11–16. Closed triangles show the result of patient number 10. (b) Dose variation vs. time of a single respiratory cycle. Dose variations were obtained from dose measurements 10 times with the micro chamber.

Figure 3 shows dose variations measured with different NBs in the same plan. Figure 3(a) shows dose variation with increasing NBs, and Fig. 3(b) shows dose variations with increasing time of a single respiratory cycle. Dose variation was decreased with increasing NBs, and NBs of >40 times maintained the dose variation within 3% in 15 cases. Patient number 10 showed dose variation more than 3%, although NBs is more than 40 times. The regularity between a single respiratory cycle and dose variation was not observed.

**Figure 4:**
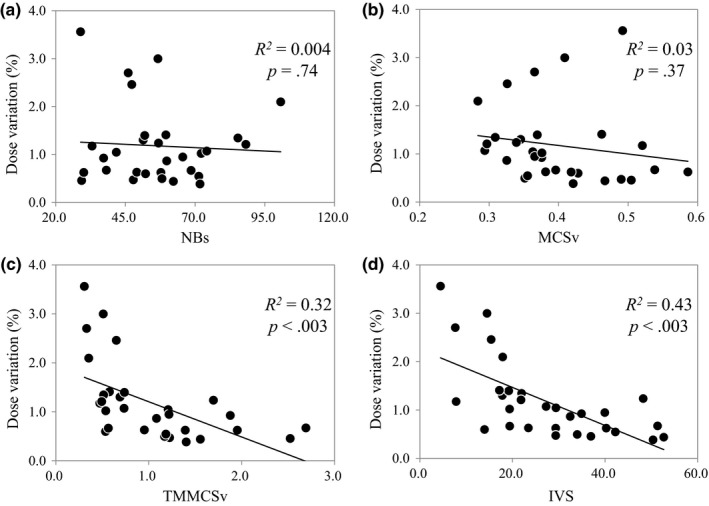
Results of correlation analysis between the plan parameters and measured dose variations. (a) NBs (number of breaths during irradiation). (b) MCSv (modulation complexity score applied to VMAT). (c) TMMCSv (the MCSv/amplitude quotient). (d) IVS (interplay effect variable score; the MCSv/amplitude quotient × NBs product)

Results of correlation analysis between the considered parameters and calculated dose variations are shown in Fig. 4. The coefficients of determination were approximately 0.43 (*P *<* *0.05) and 0.32 (*P *<* *0.05) for IVS and TMMCSv, respectively, which indicated the increasing IVS and TMMCSv are associated with lower dose variation. The NBs and MCSv showed no correlation with dose variation (*R* ^*2*^ = 0.004, *P *=* *0.74 and *R* ^*2*^ = 0.03, *P *=* *0.37, respectively).

## 4 DISCUSSION

The NBs during irradiation affected dose variation from the following results: (a) Dose variation was reduced to less than 3% by increasing the NBs to approximately 40 or more (i.e., number of breaths per minute ≥16 times) during irradiation except for patient number 10 (Fig. 3). There was an increased likelihood of larger dose variations when the NBs was <40 times during irradiation. (b) TMMCSv and IVS showed a correlation with the dose variation [Fig. 4(b) and 4(c)]. A stronger correlation was indicated in the IVS where NBs was considered. Court et al.^11^ concluded that the interplay effect is reduced with double arcs compared with a single arc, because the time taken to deliver a RapidArc plan averages out many of the dose errors (hot and cold dose spot) due to the interplay effect. Similarly, Ong et al.^12^ reported that single‐fraction 2400 MU/min flattening filter‐free RapidArc lung stereotactic body radiation therapy is susceptible to the interplay effect, but two arcs reduced the effect to a level that appeared clinically insignificant. Tyler et al.^6^ discussed that the interplay effect increased as the respiratory cycle was extended to nonclinical cycles of 30 and 60 s, with maximum deviations of ±18.2% and ±5.7%, respectively in the GTV dose metrics for dynamic MLC IMRT and VMAT‐SBRT treatments. Moreover, Stambaugh et al.^13^ resulted that the interplay effect was negligible for the motion amplitudes and respiratory cycles obtained from the 4DCT, but for the large motion and increased cycle (60 s), a significant interplay effect was observed, with D99% ranging from −16% to 17%. Therefore, extended beam‐on time or short respiratory cycle leads to decrease of the dose variation with increase of NBs during irradiation. The results of this study are consistent with their findings. Previous papers have reported several solutions to reduce the dosimetric impact of the interplay effect: adjusting the maximum MLC speed^14^ or dose rate.^12^ These solutions require modifications to the plans; consequently, it might restrict planning flexibility. In addition, excessive extension of irradiation time increases the likelihood of patient motion during irradiation.^15^ If these solutions were adapted for the patients who have relatively small dosimetric impact due to the interplay effect, the modification of plan operates as disadvantages. Therefore, the NBs should be managed for patients treated with VMAT‐SBRT. If the dose variation is ≤3%, NBs >40 times would be necessary in this study. The Mann–Whitney U test was used to compare difference between two groups of dose variations which were divided into based on the threshold of NBs, and it showed a significant difference (*P* < 0.001). Patient number 10 showed dose variation more than 3% while NBs was more than 40 times. The different result from other patients was caused by displacement of respiratory waveform during irradiation.

There are two methods for managing NBs during irradiation. A breath hold method^16^ avoids the dose variation related to the interplay effect. The breath hold method, however, leads to the extension of the treatment time. Also, the reproducibility of the breath hold phase is uncertain. In contrast, the audio coaching method^17^ does not extend the treatment time or cause uncertainty about the reproducibility of the breath hold phase. For patients who breathe <40 times during irradiation, the audio coaching using a metronome may increase the patients’ NBs to achieve >40 breaths, reducing the dose variation due to the interplay effect to insignificance.

Some studies^14^, ^18^, ^19^ investigated whether plan complexity caused the dosimetric impact of the respiratory motion in the VMAT, and found that the interplay effect was greater for more modulated plan. Ehrbar et al.^20^ concluded that the interplay effect was not correlated to the modulation factor. In the present study, the correlation between MCSv alone and the dose variation was not found. The MCSv was shown an average of 0.4 ± 0.1, and the complexity of MLC sequence for 30 plans was different slightly. The MCSv may indicate similar value in the plans which are created for the same conditions such as the site, prescription dose, and number of arcs. Therefore, MCSv alone may lack to evaluate the dose variation caused by the interplay effect, even if the MLC sequence was evaluated quantitatively.

There are limitations to this study. Respiratory waveforms were obtained by external motion. It should be acknowledged that this does not necessarily correspond perfectly to internal motion. The amplitude was measured only in the craniocaudal direction, and did not consider account 3D tumor motion. Because 3D tumor motion is likely to further complicate dose variation, it is necessary to consider it in future studies on this subject. The NBs depends on the patient's respiratory period and the irradiation time. Irradiation time is not known until after a treatment plan is created. Therefore, it is not known until after a plan has been made whether that patient is suitable for VMAT‐SBRT. This means there would potentially have to be a new treatment plan created. Cutoff breaths >40 times is only applicable for the specific conditions used here (dose rate, Varian VMAT leaf motion, dose per fraction, the number and length of arcs, etc.). More data may be required to ensure determining a patient's suitability for lung VMAT‐SBRT.

## 5 CONCLUSIONS

The NBs is an important factor to reduce the dose variation caused by the interplay effect with VMAT‐SBRT for lung cancer. The patient who breathes >40 times during irradiation of two partial arcs VMAT (i.e., NBs = 16 breaths per minute) may be suitable for VMAT‐SBRT for lung cancer.

## CONFLICT OF INTEREST

The authors report no conflicts of interest.
